# 534. Post-burn Neck Contractures: A Systematic Review

**DOI:** 10.1093/jbcr/irag033.231

**Published:** 2026-04-06

**Authors:** Divya Hegde, Dhaval Bhavsar

**Affiliations:** The University of Kansas Health System Burnett Burn Center, Shawnee, KS; The University of Kansas Health System, Kansas City, KS

## Abstract

**Introduction:**

Post-burn neck contractures, characterized by restricted movement due to scar tissue formation, significantly impact both function and aesthetics. Numerous treatment options exist, including surgical interventions, physical therapy, and assistive devices. However, the relative effectiveness of these treatments remains unclear, particularly in improving neck mobility and reducing long-term complications. This systematic review aims to evaluate and compare the effectiveness of various treatment modalities for post-burn neck contractures, focusing on range of motion (ROM), pain reduction, and functional improvement.

**Methods:**

We conducted a systematic search across databases including PubMed, CINAHL, and Cochrane from January to March 2024. The inclusion criteria encompassed randomized controlled trials (RCTs), observational studies, and case series examining interventions for post-burn neck contractures. Outcome measures included ROM, pain scores (Visual Analog Scale), scar recurrence, graft take, and complication rates. Data were analyzed following Cochrane protocols and PRISMA guidelines.

**Results:**

A total of 28 studies met inclusion criteria, covering both surgical and non-surgical interventions. Surgical methods, particularly the use of free flaps, demonstrated the most significant improvement in ROM and patient mobility, with lower rates of scar recurrence compared to skin grafts and tissue expansion techniques. Non-surgical interventions, while less effective in severe cases, showed moderate improvements in ROM and pain reduction. The lack of standardized outcome measures across studies presented challenges in direct comparison.

**Conclusions:**

The variability in study designs and outcome measures highlights the need for standardized metrics in future research to better assess and compare treatment effectiveness. Long-term follow-up studies are recommended to evaluate the durability of these interventions.

**Applicability of Research to Practice:**

The lack of standardized outcome measures makes it difficult to compare treatments and assess long-term effectiveness. Establishing consistent metrics, especially for range of motion and scar quality, is essential for improving clinical decision-making and advancing research in post-burn neck contracture management.

**Funding for the study:**

N/A.

